# How should we evaluate the impact and implementation of palliative care education programs? The Pallium Canada experience

**DOI:** 10.1177/26323524261479750

**Published:** 2026-08-26

**Authors:** Jose Pereira, Shera Hosseini, Christopher A. Klinger, Holly Finn, Jonathan Sherbino, Michelle Howard

**Affiliations:** 1 Faculty of Medicine, University of Navarra, Spain; 2 Institute for Culture and Society, University of Navarra, Spain; 3 Pallium Ottawa, Canada; 4 Department of Family Medicine, 3710 McMaster University, Ontario, Canada; 5 McMaster Health Education Research, Innovation and Theory (MERIT) Centre, 3710 McMaster University, Ontario, Canada

**Keywords:** palliative care, education, evaluation, research, environmental scan, critical appraisal

## Abstract

Considerable effort and resources are invested in developing, implementing, and sustaining educational programs in palliative care, making evaluation an important component of these programs. Several frameworks, models and approaches are available to palliative care educators to inform their evaluation, and accompanying research activities. This paper describes the process used to develop a practical evaluation framework for Pallium Canada’s educational programs. The objective was not to produce another comprehensive review of evaluation frameworks, as several reviews already existed, but rather to identify a practical and fit-for-purpose evaluation approach that could be implemented rapidly across Pallium Canada’s expanding educational initiatives. A multiphase, multipronged approach was used, which included an environmental scan and input from a panel of national and international education and evaluation experts. Several trends in education evaluation were identified, including: a need to a) evaluate impact beyond learner feedback and acquisition of competencies, b) evaluate implementation alongside impact and outcomes, and c) approach education evaluation from a complexity lens. Twenty-one frameworks (including models and approaches) were shortlisted (and are summarized in the paper). They are organized in four categories based on their focus: a) Impact; b) Implementation; c) Economic evaluation; and d) Combinations. Several additional concepts, including pragmatism, quality improvement, and theories of change serve as useful lenses with which to approach evaluation. The final framework incorporates two main frameworks, namely the New World Kirkpatrick Model and the Consolidated Framework for Implementation Research. It also draws elements from other frameworks and approaches.

## Plain language summary

Pallium Canada, a foundation in Canada that builds primary palliative care capacity, developed a new research evaluation plan in 2021 for its education interventions. This article describes the development of this framework, which included an environmental scan of existing frameworks and approaches and was informed by 15 Canadian and International education, evaluation and palliative care experts. Pallium Canada's plan includes two frameworks, namely the New World Kirkpatrick Model and elements of the Consolidated Framework for Implementation Research; it therefore includes evaluation of both impact and of implementation. The framework has been applied in recent studies by Pallium Canada. The article also provides readers a summary of the different frameworks and approaches identified in developing Pallium Canada’s framework.

## Introduction

Education across the learning cycle, from undergraduate and postgraduate training to continuing professional development (CPD), is considered one of the main strategies to improve access to palliative care.^1–3^ Considerable effort and resources are invested in developing, implementing, and sustaining educational programs in palliative care, making evaluation an important component of these programs.^4^

Evaluation has several overarching goals, including guiding continuing improvements of a program and determining its impact. In addition to understanding the impact on learners, patients, and healthcare systems, it should identify and understand the facilitators and barriers to implementation, sustainability, spread and scale-up.

Many different evaluation frameworks, models and approaches are available to educators. They enhance the robustness and efficiency of evaluation strategies, as well as the interpretability of the results.^5,6^ The growing number of these frameworks and approaches poses both opportunities and challenges for educators as they need to identify those most germane to their respective programs.

Pallium Canada, a registered foundation and charitable organization that was established in 2001, builds primary-level palliative care capacity across different professions, care settings, and disease groups.^7^ It does this mainly through its Learning Essential Approaches to Palliative Care courses (LEAP) courses and, from 2021 to 2025, a new program that used the Extension for Community Healthcare Outcomes (ECHO) approach.

The main goal of the LEAP courses is to equip healthcare providers across different professions, services, and settings with the core competencies to deliver a palliative care approach, also referred to as primary- or generalist-level palliative care.^8^ Other goals include to promote inter-professional teamwork, to enhance collaboration between healthcare services and local specialist palliative care teams, and to stimulate palliative care-related quality improvements in the health care systems. There are currently eighteen different versions of LEAP courses, several of which target specific care settings or disease groups. There are also different versions for different delivery methods, including fully in-person, fully online and blended versions.

In 2025, 737 courses were delivered to 8550 healthcare professionals across Canada. Pallium Canada has an online Learning Management System (LMS) that supports registration and administration of all courses and learners and provides access to course materials, including learning materials and standardized evaluation questionnaires. Through the LMS, learners submit pre- and post-course knowledge, attitudes and comfort questionnaires, course evaluation surveys and post-course commitment to change statements and reflections. Pallium Canada has also promoted the development of compassionate communities across Canada.

Pallium Canada’s Palliative Care ECHO Project was a five-year national initiative (2021-2025 inclusive) funded by Health Canada in which Pallium Canada collaborated with 14 Hub Partners across Canada.^9^ The project served as a catalyst for cultivating communities of practice (CoPs) and advancing continuous professional development across care settings. In the first three years, for example, over 300 webinars were presented, of which about 50% were organised and delivered by Pallium Canada and the remainder by the partner hubs. Seven CoPs were supported by Pallium Canada’s main hub, and several others by the partner hubs.

Over the years, Pallium Canada had used various evaluation approaches.^10^ Between 2003 and 2007, for example, it drew on Health Canada’s Participatory Evaluation Framework, the Logical Framework and Kirkpatrick’s Evaluation Model.^11–15^ The evaluations had largely, with some exceptions,^16^ focused on Kirkpatrick’s levels 1 and 2, namely exploring learner experiences (satisfaction) and impact on knowledge, attitudes, comfort levels and commitment-to-change across different professions.^17–19^ Signals of impact at level 3 (patient care) and level 4 (health system impact) had been noted but had not been extensively studied. In late 2019, Pallium Canada decided to revisit its evaluation approach.

This article is a reflection on the process that Pallium Canada adopted to develop a fit-for-purpose evaluation framework and a description of the framework that emerged from the process. It also provides palliative care educators with a concise ready-to-read resource of various evaluation frameworks and approaches that they may consider when undertaking a similar process or when selecting an evaluation framework.

## Framework development process

**Figure 1.**
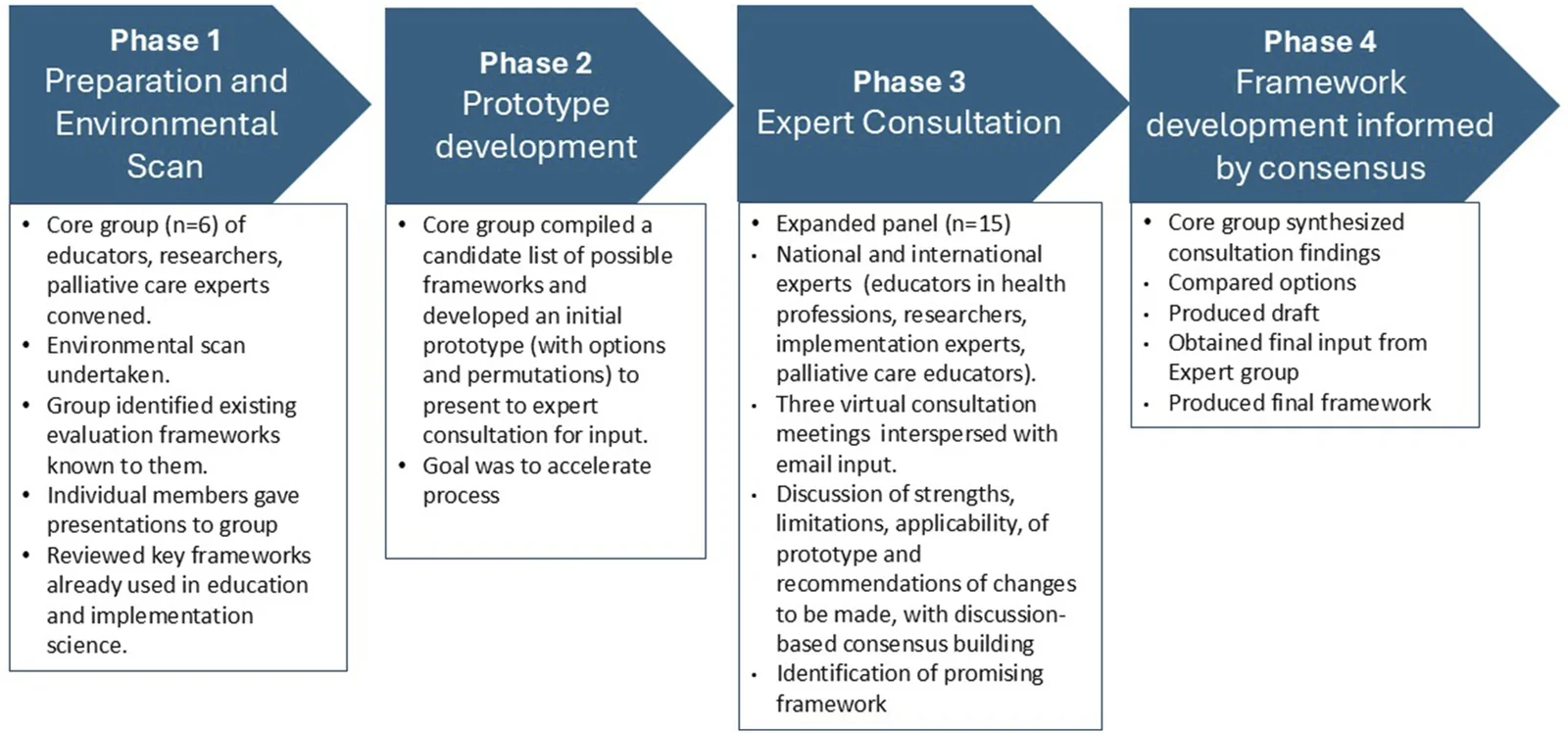
Process of developing Pallium Canada’s new evaluation and research plan (framework).

An expert-informed framework development process that consisted of four phases and used environmental scanning, stakeholder consultation and iterative consensus was applied (See Figure 1). These phases included: 1) Preparation and Environmental Scan; 2) Early Prototype development; 3) Expert consultation; and 4) Final framework development by informed consensus.

### Context and rationale

As Pallium Canada was about to start the process in early 2020, the COVID-19 pandemic was declared, and the organization had to refocus its attention and resources into undertaking the required pivoting from classroom learning to full online delivery of its courses and activities. As it recalibrated, it also became apparent that a new strategy to augment the LEAP program was needed to meet new, rapidly emerging needs in the healthcare system and amongst care providers. To this end it started a series of custom-made webinars and online workshops in March 2020. These covered topics such as re-arranging home palliative care, managing severe dyspnea and non-invasive ventilation support, reorganizing hospital and home palliative care services, dealing with loss on a large scale and self-care.

Moreover, in late 2020 Pallium Canada applied for funding to initiate a new ECHO-based program to complement its LEAP program, specifically to support communities of practice that would provide opportunities for LEAP alumni to continue learning in this area and that would address specific healthcare service needs such as integrating palliative care into advanced non-cancer illnesses and in psychiatry, among others. Funding was received in April 2021, requiring a rapid launch of this new large program. The question “How will we evaluate LEAP, ECHO and the COVID-19 webinars?” arose.

Given the urgent need to evaluate rapidly expanding national educational initiatives during the COVID-19 pandemic, and to evaluate programs post-pandemic, the objective was not to undertake another comprehensive evidence synthesis, but rather to identify a practical, evidence-informed evaluation approach suitable for immediate implementation and tailored to Pallium Canada’s needs and context. A rapid literature scan in late 2019 identified that several reviews and guideline papers already existed. What was needed was a practical and fit-for-purpose evaluation approach that could be implemented rapidly across Pallium Canada’s expanding educational initiatives. This process was consistent with how many healthcare organizations make strategic evaluation decisions in practice, particularly during periods of rapid change such as the COVID-19 era.

Rather than undertaking another formal scoping or systematic review of existing frameworks, it was felt that an environmental scan approach would be more efficient for the goal at hand and would be more sensitive to capturing emerging trends and tacit knowledge held by experts in the field. What emerged was a stakeholder-informed framework development process that included educators, researchers, implementation experts, and palliative care experts, working together to design a new framework, drawing upon the existing literature and their respective experiences and expertise.

### Environmental scan and early prototype development

The environmental scan (ES) approach is a recognized and valuable tool in health care that can provide planners, policymakers and stakeholders with knowledge and insights about current contexts (including social, economic, technological, political), and emerging trends.^20,21^

A core group (n=6) of educators, palliative care experts and researchers (authors of this article) was convened, followed by an environmental scan. This included: a) a narrative review of the literature – specifically scoping and systematic reviews on topics related to evaluating learning, educational interventions and education programs in the health professions, and their respective references – and two books identified by some members of the core team as being foundational^4,22^; and b) sharing of experiences and tacit knowledge held by member of the core group. The group was provided an in-depth orientation to Pallium Canada and its various education programs as well as the evaluation approaches it had used previously. Group members took turns presenting structured presentations to the group in which they shared past and current experiences with evaluation, including with different evaluation frameworks and approaches they had used or considered, and proposed frameworks and approaches to use.

Throughout the process the terms, *frameworks* and *approaches*, were considered separate and distinct concepts, where the former refers to a general way of thinking about or conducting an evaluation, while the latter refers to a structured set of concepts, domains, or categories that guides what should be examined and how findings should be organized. Approaches include the overall philosophy, perspective, or strategy and examples include realist evaluation, participatory evaluation and programmatic evaluation in competency-based medical education. Realist evaluation, for example, assumes that interventions and programs work differently in different contexts and seeks to understand what works, for whom and under what circumstances. Evaluation frameworks, on the other hand, are practical structures that organize key concepts and guide evaluation planning, implementation, and interpretation. They address the questions “what should be measured?”, “what domains are important?” and “how should findings be organized?”. Examples include Miller’s Pyramid, Moore’s Framework and Kirkpatrick’s Evaluation Framework.^4,23–26^

Guiding principles were developed by the core team in partnership with Pallium Canada to ensure that the future framework aligned with Pallium Canada’s programs, mission, values and funder-reporting requirements (see Table 1).

**Table 1.** Guiding Principles for Pallium Canada's Evaluation and Research Plan.

1. Meaningful and relevant to diverse stakeholders:
a. Pallium Canada (for quality assurance, quality improvement, program planning and deployment, spread and scale, etc.);
b. Palliative care stakeholders (i.e., funders, policy makers, educators, patients, and families);
c. The palliative care scholarly community.
2. Draw upon existing frameworks and approaches recognized by experts to be valid and rigorous.
3. Overall, the Plan must be pragmatic, feasible, and doable within allotted resources.
4. Flexible and allow integration of components from other evaluation frameworks or approaches if these are deemed relevant but not incorporated in a particular framework (i.e., allow some tailoring for specific cases and emerging needs).
5. Use frameworks that incorporate complexity, recognizing that education interventions like LEAP are often only one component of multi-pronged strategies and are influenced by many internal and external factors (complexity).
6. Recognize and build on previous evaluation and research on Pallium initiatives.

The core group, informed by the environmental scan, developed an early prototype of a potential framework. This early prototype included the draft of the guiding principles, a list of needs to be evaluated by the large expert group, potential options in terms of frameworks and approaches, and specific questions to ask of the larger expert group in Phase 3. This was to increase efficiency of the process; instead of having the expert panel construct a framework and identify guiding principals from scratch, they could focus on providing input, adding to the environmental scan and modifying and fine tuning the early prototype.

### Consensus-based framework development

A larger expanded expert panel (n=15) was convened, to include national and international experts, including educators in health professions, researchers, implementation experts, palliative care educators). They were also given an in-depth orientation to Pallium Canada, its LEAP and ECHO programs, and its courseware, and asked to review the early drafts of the guidelines and the prototype and to provide their expert opinions on these. This process involved three virtual meetings of the core and large expert group, interspersed with asynchronous discussions and input via email. The discussions related to strengths, limitations, applicability, and considerations of the various frameworks and approaches available relative to the proposed prototype. The prototype underwent further modifications based on the input and, through an iterative process of discussion-based consensus building, the final framework emerged.

## Insights from the process

### Trends

Several trends in education evaluation, including those relevant to programs such as LEAP and ECHO, were identified. These were: a) including the learner experience and the acquisition of competencies, but also needing to evaluate impact on patients and the healthcare system; b) a growing recognition of the need to evaluate processes, implementation, spread and scale-up (and not only impact on learners, patients and the healthcare system); and c) approaching education evaluation from a complexity lens where education interventions are usually complex and done within complex systems; one evaluation framework may not be applicable to another context or need.

The process elicited, among others, reflection and discussion on what constitutes best evidence related to education interventions. Best Evidence Medical Education (BEME) provided a useful starting point; BEME is an international initiative and collaboration that promotes basing teaching practices, curriculum design, and student assessment on the best available scientific research, rather than solely on tradition or opinion.^27^ Within BEME, systematic reviews of randomized controlled trials (RCTs) and individual RCTs, followed by case-control studies, are often considered the best level of evidence in medical education. However, RCTs, remain limited in medical education. While they are possible,^28^ they face ethical challenges and the complexity of interventions and real-world variability make them difficult to implement. As a result, researchers often turn to alternative study designs like cohort studies, qualitative research, and quasi-experimental designs to provide valuable insights into the effectiveness of educational interventions.

#### Evaluating impact beyond learner feedback and acquisition of competencies

Evaluations of education programs often focus on learner experiences and the acquisition of different competencies. Evaluations of impact on patients, the healthcare system and return on investment (ROI) are less common.^29^ This is understandable as evaluations that explore these constructs generally require methods that need more resources, expertise, and complex data sources. Notwithstanding the challenges, they are important constructs to assess and increasingly encouraged by policymakers, funders, scholars, and academic journals. This rising focus parallels growing demands to curb healthcare costs and understand the value-add of health interventions.^30^

#### Evaluating processes, implementation and spread

There are growing calls for evaluation efforts that go above and beyond outcomes and impacts and consider contextual and process-oriented factors that enhance or impede learning, implementation, uptake and spread.^31–36^ The conditions and processes that impact implementation are, for example, the focus of the Consolidated Framework for Implementation Research (CFIR).^37,38^ The updated Kirkpatrick Model – the New World Kirkpatrick Model (NWKM) – now also includes some of these constructs, specifically strategies that reward, encourage and reinforce learning.^30^ Van Melle and colleagues propose a movement from attribution analysis, that looks for direct causal relationships between the program activity and outcomes, to contribution analysis that focusses on the question “How much of a difference (or contribution) has the program made to the observed outcomes?”^33^

Implementation in healthcare is the process of putting evidence- or best-based practices, research, guidelines or new policies into routine use. It is the critical “how-to” phase that bridges the gap between discovering what works in the research setting and delivering that proven care, approach or product to the everyday real world. It involves the selection and deployment of implementation strategies to enhance uptake of an effective practice or treatment. Implementation of innovations into practice can be a complex process.^31,32,39^

A science has evolved on how best to enhance implementation, from smoking cessation interventions – where questions are asked about what the most effective strategies could be to result in smoking cessation – to how to spread and scale-up an intervention or practice that has shown positive impact. Implementation science takes a structured and phased approach to developing, replicating, and evaluating an intervention in multiple sites and contexts.^40^ It seeks to understand and work within real world conditions, rather than trying to control for these conditions or to remove their influence as causal effects.^34^ Context plays a central role in implementation research and can include the social, cultural, economic, political, legal, and physical environment, as well as the institutional setting. It often includes various stakeholders and their interactions.^41,42^

For Pallium Canada, implementation is particularly relevant with respect to pedagogical (andragogical) strategies that enhance application into practice of what is learned in the LEAP courses or ECHO sessions, and with respect to how best to spread (replicating an intervention) and scale-up (building infrastructure to support full scale implementation) the initiative across different contexts such as professions, care settings, and jurisdictions. Spread and scale-up can be difficult and are subject to many influences and variables from context to context.

Implementation and implementation science draw from various fields, including complexity and social sciences, where the former encourages a flexible and adaptive approach to change given the complexity of healthcare systems and the latter considers why people act in the way they do, especially the personal, social, and forces that shape people’s actions, or lack of action. For example, the adoption by clinicians of a new healthcare innovation can be challenging; changing how clinicians and care providers act, think, and deliver daily services is not necessarily easy or assured.^40^

There exist many implementation-related frameworks and approaches to select from, ranging from Logical Frameworks (logFrames) to the Realist Model.^13,34,43^ There are also many cognitive, social and behavioralist theories of change that are pertinent to implementation, from Roger’s Diffusion of Innovation to the Theory of Planned Behaviour. Moullin and colleagues explain that this variety, and sometimes disparity, of implementation frameworks is because they originate from different disciplines, professions, philosophies and contexts.^36^ Implementation concepts, they say, are designated as including the process of implementation (divided into a series of phases, stages or steps), domains (groups or levels of influences), and three elements: factors (barriers and enablers), strategies and evaluations.

Therefore, as implementation frameworks vary in their orientation, it is plausible, by design or otherwise, that not all frameworks targeting a particular innovation cover all implementation concepts. Selecting an implementation framework can therefore be a complex undertaking. When an organisation or healthcare provider seeks to implement an innovation, an important consideration is whether a framework specifically developed for that innovation is the most appropriate choice, or whether an existing framework—or combination of frameworks—originally designed for other innovations may provide a better fit for the implementation context.

#### Evaluation that recognizes complexity

Education, like public health and social interventions, often includes interventions with several interacting components, which in turn are influenced by many extrinsic and intrinsic factors.^41,42^ They are undertaken within healthcare systems that are themselves complex.^43^ In addition to differentiating between the relative role of each of the components, there are additional challenges such as the difficulty of standardising the design and delivery of the interventions across different contexts. It is sometimes difficult to attribute changes to specific programs given the multiple confounding variables in complex environments.

This calls for more realist-based approaches.^34,44^ Methods such as randomized controlled research methods, commonly valued in healthcare, may not capture the context of complex, real life situations. For fluid and complex contexts, Poth and colleagues call for more reflective approaches that strive to understand what initiatives are doing rather than attending to inflexible protocols and pre-determined outcomes.^45^

### Frameworks

Several frameworks were deemed potentially pertinent for Pallium Canada’s LEAP and ECHO programs and shortlisted for consideration (Table 2). Summaries of these are provided in Supplement A. They are organized into four categories: A) Impact evaluation; B) Implementation evaluation; C) Economic evaluation; and D) Combinations of these.

**Table 2.** Shortlist of evaluation models, frameworks and approaches deemed most germane to Pallium Canada’s LEAP and ECHO Programs*.

**Evaluate impact**
1. Miller’s Triangle^46–48^
2. Moore’s Model (Seven Levels Outcomes Model)^24^
3. Kirkpatrick’s Model^25,49,50^
4. Modifications to the original Kirkpatrick Model: Phillips’,^51^ Stokking,^49^ Kaufman and Keller,^52^ Hammick et al.,^53^ and Barr^54^
5. Kirkpatrick’s New World Model (NWKM)^30,50^ (includes drivers of change)
**Evaluate processes, implementation, and spread**
• Consolidated Framework for Implementation Research (CFIR)^37^ [https://cfirguide.org/]**
• Developmental Evaluation (DE)^44,55–57^ **
• Active Implementation Framework^58^ **
• The RE-AIM (Reach, Effectiveness, Adoption, Implementation, and Maintenance) Framework to Assess community-based Programs^59–61^ **
**Evaluate economic impact and return on investment (ROI)**
• Health Technology Assessment (HTA)^62^
**Evaluate combinations of impact, processes and/or ROI**
6. Kirkpatrick’s New World Model (NWKM)^30,50^ **
7. The Medical Research Council’s Framework^41^ **
8. Realist Model^34,63^ **
9. Contribution Analysis (CA)^33,64^ **
10. Logical Framework^13^
11. 3-Wishes Project Evaluation Framework^65^
12. Stake’s Countenance Evaluation Model^66^
**Lenses, Considerations and Approaches** * * *
13. Complexity^42,67^
14. Quality Improvement and the Triple Aim,^68^ Quadruple Aim^69,70^ and the Six Dimensions of Quality^71^
15. Participatory Research,^72^ Community-Based Participatory Research (CBPR),^73,74^ and Action Research^74^
16. The Pragmatism Paradigm^75,76^
17. Social Network Analysis (SNA)^77–79^
18. Theories of Change
• Theory of Planned Behaviour^80–82^
• Michie et al.’s Behaviour Change Wheel^83,84^ and the COM-B-System^83^

*With Pallium Canada’s LEAP and ECHO Programs as examples.

**Particularly suited for complex interventions and contexts.

***Those deemed relevant to programs such as Pallium Canada’s LEAP and ECHO Programs.

The Consolidated Framework for Implementation Research (CFIR), is a conceptual evidence-derived framework to guide systematic assessment of multi-level implementation contexts to identify factors that might influence implementation and effectiveness.^37,38,85^ The Developmental Evaluation,^44,55,56^ the Active Implementation Framework,^58^ the Realist Model,^34,63^ Contribution Analysis,^33,64^ and the RE-AIM (Reach, Effectiveness, Adoption, Implementation, and Maintenance) framework^59–61^ also focus on evaluating implementation and processes. All are well suited for complex interventions and contexts. The RE-AIM Framework is especially applicable for community-based programs.^60,61^

The Development Evaluation (DE) approach draws on insights about complex dynamic systems, uncertainty and nonlinearity, and is particularly useful within fluid contexts.^44,55–57^ Educators can study, document and make sense of changes that occur (including unexpected ones) as the program is implemented. Patton stresses its utility in the evaluation of complex, emergent initiatives in the early stages of development.^44^ Reflective approaches – such as the DE – are needed to respond to what initiatives are doing rather than attending to pre-determined outcomes.

Frameworks such as Moore’s Seven Levels Outcomes Model,^24^ Kirkpatrick’s Model,^11,25,49,52^ and Kirkpatrick’s New World Model (NWKM)^30,50^ primarily focus on outcome evaluation and include different levels of evaluation, ranging from reach (number of learners) and changes in learner knowledge and other competencies, to impact on patients and the healthcare system. The NWKM, a more recent update on the Kirkpatrick model, includes aspects of implementation evaluation, including drivers of change such as (incentives and conditions).

The Realist Model, which often uses mixed methods research approaches, goes beyond assuming a simple cause-and-effect association and recognizes that an educational intervention may provoke different reactions and results in different learners, contexts and even times.^34,63^ Wong and colleagues stress that both context and mechanism must be systematically researched along with intervention and outcome.^34^

The different models have their respective strengths and limitations.^50,64,86^ These are relative and depend on the context and needs of the evaluation; a strength in one context may represent a limitation in another (See Supplement A).

There is considerable overlap across some of the models and frameworks regarding the levels of evaluation and constructs they include. In terms of impact on learners’ knowledge, attitudes, and self-efficacy, for example, there is overlap between the NWKM and Moore’s Model.^24,50,87^ The Realist Model poses the question “What works, for whom, in what circumstances, in what respects, to what extent, and why?”,^34,63^ while the Contribution Analysis Model asks “How much of a difference (or contribution) has the program made to the observed outcomes when multi-pronged approaches are used?”.^33^

Some models and approaches were not designed for education but are transferrable to educational contexts. The RE-AIM,^59–61^ the Active Implementation Framework,^58^ and Developmental Evaluation (DE) are examples.^44,55–57^

Some frameworks, such as Kirkpatrick’s models, provide guidance on constructs and levels, but do not specify the outcomes to assess, methods to use and the sources of data. The onus is on educators to determine these, relying on various factors to inform their choices, including resource and expertise availability and ease of access to data. Others, such as Moore’s model and DE, are more specific. The DE, for example, suggests approaches such as service and care provider interviewers, stakeholder interviews and surveys, patient and stakeholder interviews, program data, and document and chart reviews.^44,56,88^ Methods to assess learner competencies in undergraduate and postgraduate education have previously been elucidated.^89,90^ Generally, the outcome measures and data collection methods should align with the frameworks’ levels and constructs, as well as available resources and evaluation goals. Sources of data for higher level evaluations, for example, could include administrative claims, electronic and medical health records, healthcare provider surveys, and patient surveys.

There is flexibility in the application of most frameworks. In the case of the CFIR, for example, users are instructed to select the constructs that best meet their needs, circumstances, and contexts.^37,38,85,91^

### Lenses and other approaches

There are several approaches or lenses that, although not formal models or frameworks, help educators design and implement their evaluation plans. These include Quality Improvement and its related six aims (safe, timely, effective, efficient, equitable and patient-centered or STEEP) of the Institute of Medicine^71^ and the Triple,^68^ Quadruple^69,70^ and Quintuple Aims.^92^ The Quintuple Aim adds equity to healthcare to the aims of improved patient experience, improved quality of care, improved efficiencies and improved care provider experiences.^92^ Other approaches or lenses include the Pragmatism Paradigm,^75^ Social Network Analysis,^77–79^ Theories of Change*,* Participatory Action Research and Community-Based Participatory Research.^72–74,93^ See Supplement A for summaries.

### Evaluation and research framework for Pallium Canada

Several overall recommendations emerged from the expert engagement for the new framework. These included:

• Pallium Canada should attempt to evaluate both impact and implementation (including what works, when and how, contexts and processes).
• Evaluating the impact and implementation of LEAP and ECHO programs requires multiple evaluation and research activities (sub-studies). Evaluation and research are closely aligned; some evaluation activities require the use of research methods.
• Three approaches should be recognized with respect to who conducts the evaluation and research of the LEAP and ECHO programs: a) conducted by Pallium Canada alone (*Internal*); b) undertaken by Pallium Canada in collaboration with partners (*Partnership*); and c) undertaken by third parties independent of Pallium Canada (*Third-party*).
• Any LEAP and ECHO activity related to Indigenous Peoples should be led and informed by Indigenous persons, consistent with the guiding principles of Ownership, Control, Access, and Possession (OCAP) that includes Indigenous knowledge.^94^ Participatory Action Research (PAR) and Community Based Participatory Research provide useful approaches in this area.^95^

**Figure 2.**
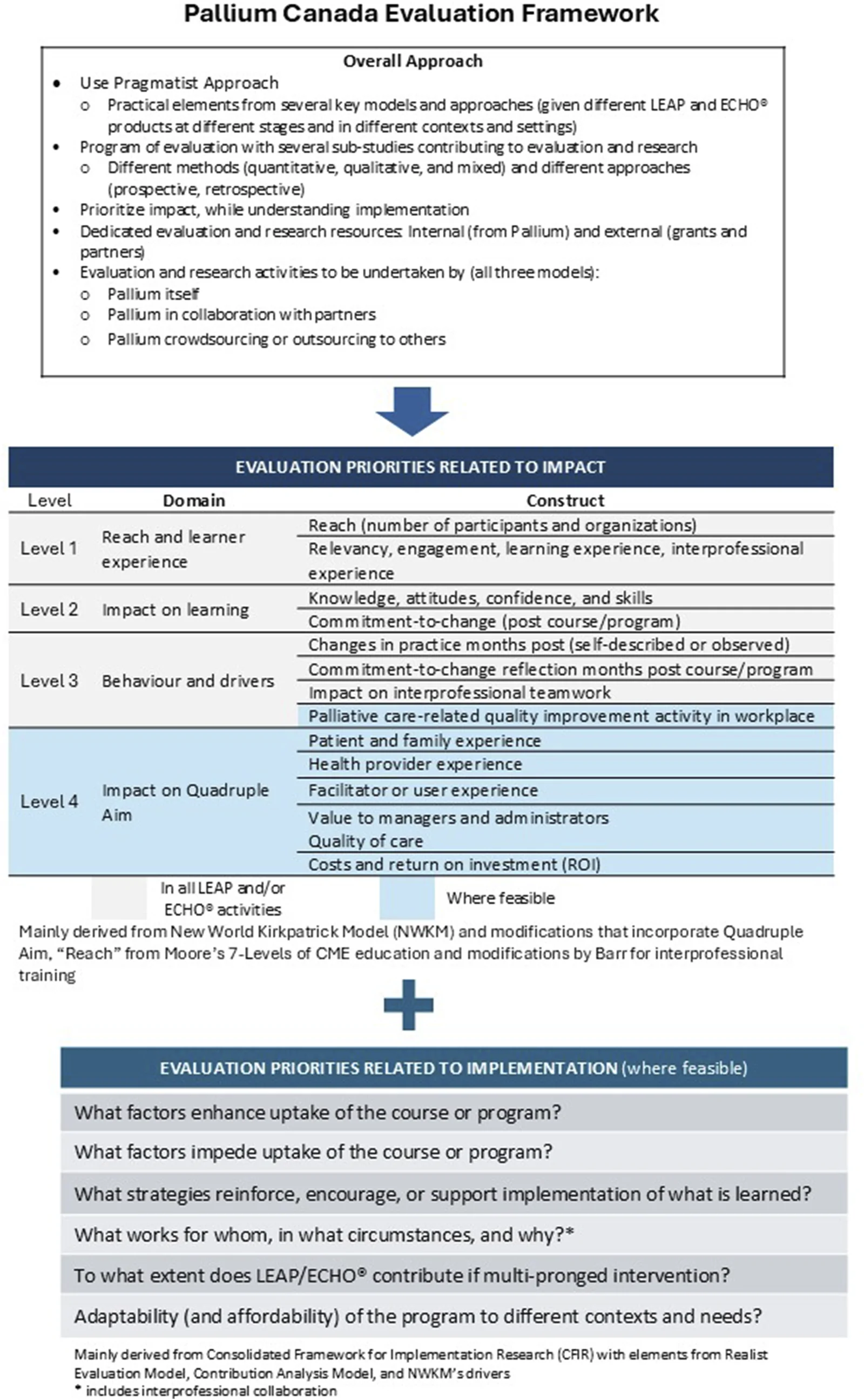
Pallium Canada LEAP and ECHO programs evaluation framework.

The framework is shown in Figure 2. It is divided into three boxes and leverages two existing frameworks while drawing on some constructs from other frameworks and approaches. The first box lists key considerations and overall approaches, the second describes the priority constructs related to evaluating outcomes (largely drawn from the NWKM), while the third describes constructs related to evaluating implementation (largely drawn from the CFIR).^30,38,50,85^

The NWKM was chosen as the model to inform impact (outcomes) evaluations and highlight drivers, while the CFIR was chosen for its relevance to evaluating implementation. Adopting these two frameworks allow Pallium Canada to leverage existing reputable and empiric-based frameworks and build on approaches Pallium Canada has previously used and allow the two to complement each other; one focussing on impact and the other on implementation. While “Impact” and “Implementation” evaluations may be done simultaneously, one or the other may predominate at times. For example, in a new ECHO COP the focus may initially be on implementation instead of impact.

These frameworks are augmented by elements from other frameworks, approaches and lenses. To the NWKM, we added “reach” from Moore’s Framework to Level 1, as well as some aspects of interprofessional learning evaluation proposed by Hammick et al.^53,96^ Phillips’ economic assessment construct was integrated into Level 4.^51^ As leading indicators and desired outcomes for Level 4, we adopted constructs from the Quadruple Aim^69,70^ and the concepts of value, transferability, and sustainability from the 3-Wishes Project,^65^ a program for dying patients in intensive care settings, as these are well aligned with the LEAP and ECHO programs.

While evaluations of satisfaction levels, or changes in knowledge and other competencies are insufficient,^50,64,86^ the learner experience remains a necessary component of any evaluation.^5^ Although good satisfaction ratings do not necessarily guarantee learning, bad ratings may decrease the probability of it occurring and may undermine promotional efforts.

Only those domains and constructs from the CFIR deemed most pertinent to the LEAP and ECHO programs are incorporated in the plan (see Figure 2). We selected them based on priority, relevancy, and practicality.

The use of these two frameworks, and drawing elements from other approaches, provides Pallium Canada with the flexibility needed to undertake evaluations across different products, contexts and stages of development. There may also arise situations where a funder or partner may require a specific evaluation framework or approach such as the Logical Framework or RE-AIM Framework. In such cases, the overall framework is flexible to adopt the approach or to inform the development of the logical framework.

For each level and construct in the plan, specific research questions were identified, as well as possible study methods and data sources (see Table 3). Many of the data sources and collection methods, infrastructure and processes are already in place, including the standardized and validated pre/post surveys and questionnaires, commitment-to-change approach,^97,98^ and Pallium Canada’s LMS. Given the growing partnerships, multi-site collaborations with the LEAP and ECHO programs, and data sources, additional database capacity through the LMS and online database platforms like the Research Electronic Data Capture (REDCap) has been added. REDCap is an open source, browser-based, metadata-driven electronic data capture (EDC) software and workflow platform that also includes surveying capabilities.^99–101^

**Table 3.** Research Priority questions and areas, and opportunities for research in Pallium Canada’s LEAP and ECHO programs.

**Overall:**
• For organizations and/or groups that have taken LEAP, how has LEAP impacted them (and how can we assess this impact retrospectively)?
• What could prospective evaluations of LEAP and ECHO look like?
**Interprofessional CPD**
• What works and what doesn’t work for promoting interprofessional learning and collaboration in the LEAP and ECHO programs?
• What is the impact of uni-professional versus interprofessional continuing professional development (CPD) in palliative care?
• How can the courses be fully inclusive (learning needs and scopes of practice) of all targeted professions?
**Implementation**
• What is the uptake of LEAP and ECHO across different course versions, professions, settings, contexts and jurisdictions?
• What are the factors that accelerate or impede uptake, implementation, spread and scale-up across different settings, professions, contexts, and jurisdictions?
• How does LEAP and ECHO change the way attendees practice and provide a palliative care approach? How does this vary by profession/program?
**Patient and family outcomes**
• What is the impact of LEAP training on patients and families?
• How do ECHO programs impact patients and the healthcare system?
**Patient, Care and Healthcare System Impacts**
• To what extent, and in which areas and ways, do the LEAP and ECHO programs impact patients, healthcare providers, the healthcare system, managers and other stakeholders in different settings, contexts and jurisdictions? And on return on investment (ROI)?
**Other**
• How and to what extent do the LEAP course (different courses) and the ECHO programs build primary-level palliative care capacity and spread the palliative care approach?
• What is the role of comfort, confidence, and commitment to change in terms of enhancing implementation of what was learned into practice?
• Do different video formats affect learning when teaching communication skills?
• What is the role and impact of commitment-to-change in LEAP courses?
• Is there a role for the commitment-to-change approach in the ECHO programs?
• To what extent does LEAP and ECHO training impact healthcare provider experiences?
• How do LEAP’s various learning methods, delivery methods (classroom versus hybrid or flipped versus all-online), and approaches contribute to the learning experience and impact on skills acquisition and impact on patients and the healthcare system?

### Application of Pallium Canada’s framework

The new framework has informed several studies since 2021. In a study of the impact of LEAP Hospital training on the hospitalists (family physicians working within a hospital as generalists) in a small community hospital, colleagues and peer of the learners, including nurses and nurse managers who had not undertaken LEAP training but worked with hospitalists, were interviewed to explore changes in behaviour and impact on patient care that they had observed in the hospitalists following training.^102^ In an evaluation of the impact of LEAP Long-term care (LTC) training in nursing homes, aspects of implementation were explored.^103^

The framework was applied to the evaluation plan for Pallium Canada’s ECHO program. The details of the ECHO project and its components are published elsewhere.^9^ Through this initiative, a broad range of CoPs and webinars were delivered at national, provincial, and regional levels. During the project, 656 webinars were presented in total, with 39,986 attendances and over 63,000 views on YouTube. It is a complex project with different components and many different stakeholders (several partner organizations using it in different ways, different professions and disciplines) and different contexts (some communities of practice relate to clinical care in different care settings while others relate to development of services and integration of palliative care in different services). There was also specific funder reporting requirements and different data repositories (Pallium Canada and thirteen partner hubs). The framework and its guiding principles ensured various evaluations components and perspectives were included and provided the collaborating partners with a common understanding of the project's evaluation approach.

The framework is informing the evaluation and research plan of a new collaboration between Pallium Canada and a region-wide health systems network that includes several hospitals, long term care homes and primary care clinics. The initiative aims to train healthcare professionals on the palliative care approach, using the LEAP courseware, across several hospitals and their respective in-patient and outpatient services, community services, primary care clinics and nursing homes. The framework helped identity levels of impact to assess – which will include patient and health services level as opportunities to do this exist, including availability of population health administrative data – and what to consider in terms of studying implementation. Implementation is important in this project as it includes many different services, units and settings of care. Moreover, the organisation will also be complementing the training with quality improvement initiatives and evaluating these using the quadruple and quintuplet lenses.

The framework is also being used to guide several current projects, including the piloting of the LEAP courseware and approach across several jurisdictions in Spain (J Pereira, M Lama, J Chaccour-Diaz unpublished) and a large-scale workforce training in a large community-based academic hospital network in Canada. In the former, there is a specific focus on studying implementation, particularly given its application in a different context and culture to that of Canada. This includes interviews with collaborators, stakeholders, learners and other participants. The latter will use large system-level data to assess impact and mixed methods approaches to study implementation in different services and settings.

## Discussion

Multiple frameworks, models, approaches and lenses are available to inform evaluation and research of palliative care education initiatives. The flexibility inherent in some frameworks allows educators to incorporate aspects and constructs from other approaches that help fill in gaps or address program evaluation priorities and realities. This was done in the case of Pallium Canada’s new evaluation and research framework. Flexibility may however risk losing the integrity and robustness of proven approaches and may reduce opportunities to compare across initiatives. Notwithstanding, given the context of complex interventions in complex environments and different evaluation requirements, flexibility is often essential.

The large number of existing frameworks and their numerous corresponding constructs presents educators with the quandary of selecting between them. Several factors influence selection. These include the goals of the evaluation, the requirements of stakeholders and funders, and the available resources.

Educators need to choose strategically what to evaluate as it is not possible to evaluate everything. A critical question is “In this program, what are the key evaluation goals?” Although program improvements and implementation decisions are often key drivers, in some cases reputational risk and demands from funders or policymakers may need to be considered. Compromises are often needed as long as rigor and relevance are not undermined. Outcome measures and goals must be selected judiciously to ensure they are appropriate, accessible, realistic, feasible, and relevant.^50,90,104^

Research and evaluation into higher levels of education impact and implementation sometimes require additional resources, including funding. The work requires, among others, more sophisticated studies to assess impact on patients and the healthcare system and studies that explore economic impact of CPD interventions and programs (and the expertise required for that).^29,105^ Evaluations of impact at these higher levels are often elusive and few studies link costs and effectiveness of continuing medical and professional development to improved quality of care.

There are approaches to assess financial aspects of education initiatives. Ravyn et al. for example, developed a model to undertake return of investment (ROI) assessments using learners’ commitment-to-change statements to make economic impact predictions.^105^ Other strategies include a focused evaluation that concentrates on the highest priority and high stakes work. Large-scale, time intensive activities may be replaced by smaller but still valid and representative surveys and interviews with key stakeholders. Brainstorm meetings with stakeholders to co-develop pragmatic evaluation goals and solutions is essential. Among other benefits, it aligns their expectations with funding levels and program needs.

Interpreting evaluation and research findings requires prudence. Positive learner reactions may not lead to improvements in care. Complexity may cloud the interpretation of evaluation results in several ways. A lack of impact may reflect implementation failure rather than intervention design flaws. Identifying a single primary outcome may not make best use of the data; a range of measures is often needed, including unintended consequences. Third, ensuring strict adherence to a protocol may be inappropriate and adaptation to local settings may be needed.

Finally, consideration needs to be given to whether evaluation and research activities require research ethics board (REB) reviews and approval. Although program evaluation often falls with the domain of quality improvement, it is prudent to always receive the approval, or at least exemption, from a research ethics committee.^106–108^

### Limitations

We approached the project from the perspective of identifying an appropriate evaluation plan specifically for Pallium Canada’s LEAP and ECHO programs. The plan and the insights derived from the development process may therefore not be applicable for all programs and contexts. However, a goal in publishing this manuscript is to provide palliative care educators with key findings from this environmental scan and resources. Work is under way to replace the Quadruple Aim with the Quintuplet Aim in the framework’s impact level 4.

## Conclusions

There are several useful evaluation frameworks, models and approaches available to palliative care educators and researchers. We developed a pragmatic process for selecting an evaluation framework suitable for a national palliative care education organization operating under real-world constraints. This paper, by describing an evaluation and research plan for Pallium Canada’s LEAP and ECHO education programs and sharing the results of the environmental scan that informed it, provides palliative care educators with insights and strategies to develop evaluation strategies for their educational interventions. Emerging trends in evaluation science are identified and the need to evaluate various impact constructs and understand the context and factors that enhance or impede implementation and spread are highlighted.

## Supplemental material

Supplemental material - How should we evaluate the impact and implementation of palliative care education programs? The Pallium Canada experience Supplemental material for How should we evaluate the impact and implementation of palliative care education programs? The Pallium Canada experience by Jose Pereira, Shera Hosseini, Christopher A. Klinger, Holly Finn, Jonathan Sherbino, Michelle Howard in Palliative Care and Social Practice

## Data Availability

All data pertaining to this work are contained within the paper and its supplementary materials.*
