# Supplementary material for: How should we evaluate the impact and implementation of palliative care education programs? The Pallium Canada experience
Source: Palliat Care Soc Pract. 2026 Aug 26;20:26323524261479750. doi: 10.1177/26323524261479750 (PMC13519215; doi:10.1177/26323524261479750)
Supplement: Supplementary file 1 [file sj-pdf-1-pcr-10.1177_26323524261479750.pdf]

Supplement A: Summaries of Evaluation Models, Frameworks, Approaches and Lenses

	Description	Strengths* and Limitations*
**Evaluation of impact**		
**Miller's Pyramid**(74,75)	Describes 4 levels of competency in education and workplace settings:<br>Level 1: Knows (declarative knowledge)<br>Level 2: Knows how (procedural knowledge)<br>Level 3: Show how (performance)<br>Level 4: Does (behaviour in everyday practice)	**Strengths**<br>• Ease of application (not necessarily of data collection).<br>• Well established.<br><br>**Limitations**<br>• Does not specify which methods or approaches to use.<br>• Excludes impact on patient and healthcare system levels.<br>• Excludes evaluations of processes, implementation and spread.
**Moore's Seven Levels Outcomes Model**(41)	Builds on Miller's Pyramid.<br><br>Level 1: Participation (e.g. number of learners, professions, units trained, % of workforce trained in organization, etc);<br>Level 2: Satisfaction of Learners;<br>Level 3a: Learning: Declarative Knowledge (knows);<br>Level 3b: Learning Procedural Knowledge (knows how, skills);<br>Level 4: Learning: Competence (shows how; performs in education settings like simulated patients)<br>Level 5: Performance (does, behaviour in practice/workplace)<br>Level 6: Patient Health;<br>Level 7: Community Health.	**Strengths**<br>• Adds additional levels to Miller's Pyramid and higher levels.<br>• Ease of application (not necessarily of data collection).<br>• Provides examples of data sources<br><br>**Limitations**<br>• Excludes other competencies such as attitudes, self-perceived comfort or efficacy.<br>• Excludes evaluations of processes, implementation and spread.
**Kirkpatrick's Model**(11)	Describes four levels of evaluation:<br>Level 1: "Reaction" (learner satisfaction) and relevance of program and quality of content, quality of instruction<br>Level 2: "Learning" (changes in knowledge, skills, attitudes, confidence)<br>Level 3: "Behaviors" (changes in practice), degree to which learning implemented<br>Level 4: Organizational performance.	**Strengths**<br>• Ease of application (not necessarily of data collection).<br>• Flexibility allows educators to select measure or approaches for each level that is most practical and applicable to their context.<br><br>**Limitations**<br>• Does not include implementation, processes, contributions, economic and impact at society or community level.<br>• Implied causal chain in model.
**Modifications to original Kirkpatrick's Model**	Some evaluators and researchers have suggested modifications to Kirkpatrick's Model to address gaps in the model or specific applications of the model:<br>• Phillips'(76): Level 5 added to assess return on investment. (ROI)<br>• Kaufman & Keller(78): Level 6 added to assess "Impact on Society".	**Strengths**<br>• Address some gaps or limitations of Kirkpatrick's Model.

	Description	Strengths* and Limitations*
	"Implementation" added to Level 2. Added implementation component (what conditions and factors can the results be attributed to?)<br>• Stokking(77): Move implementation related domain suggested by Kaufman and Keller's from Level 2 to a higher level or chronologically later.<br>• Hammick et al and Barr(60)(79): Modifications made to each Kirkpatrick level to specifically evaluate interprofessional education.	
**Kirkpatrick's New World Model (NWKM)** (23)(43)	Similar four levels as original Kirkpatrick's Model (above), but with some additions that address some of the original model's limitations:<br>Level 1 ("Reaction") now includes "Engagement" (the degree to which participants are actively involved in and contributing to the learning experience) and "Relevance" (the degree to which participants will have the opportunity to use or apply what they learned).<br>Level 2 now includes "Confidence" (perceives that they will be able to actually apply the learned knowledge/skills) and "Commitment" (intend to apply the new learning).<br>Level 3 (behaviours) incorporates "Required Drivers" of learning such as processes and systems that reinforce, encourage, and reward performance of critical behaviours on the job<br>Level 4 (organizational performance) has "Leading Indicators" added to it, which are short-term observations and measurements suggesting that critical behaviors are on track to create a positive impact.	**Strengths**<br>• As for Kirkpatrick's Model.<br>• Modifications that address some of the original model's limitations, including evaluations of processes and implementation.<br><br>**Limitations**<br>• Evaluation of processes and implementation relatively rudimentary.<br>• Accompanying new diagrammatic depiction of model may be confusing
**Evaluation of processes, implementation, spread and scale-up**		
**Consolidated Framework for Implementation Research (CFIR)** (26)(6) **[https://cfirguide.org/]**	The CFIR is composed of five major domains, each of which may affect an intervention's implementation. There are 39 constructs within these domains:<br>• *Intervention characteristics*, which are the features of an intervention that might influence implementation. Eight constructs are included in intervention characteristics (e.g., stakeholders' perceptions about the relative advantage of implementing the intervention, complexity).<br>• *Inner setting*, which includes features of the implementing organization that might influence implementation. Twelve constructs are included in inner setting (e.g., implementation climate, leadership engagement).<br>• *Outer setting*, which includes the features of the external context or environment that might influence implementation. Four constructs are included in outer setting (e.g., external policy and incentives).<br>• *Characteristics of individuals involved in implementation* that might influence implementation. Five constructs are related to characteristics of individuals (e.g., knowledge and beliefs about the intervention).<br>• *Implementation process*, which includes strategies or tactics that might influence implementation. Eight constructs are related to implementation process (e.g.,	**Strengths**<br>• Provides educators a comprehensive, single-point compilation of factors that may influence intervention implementation and effectiveness.<br>• Organized into five, easy-to-understand, domains and corresponding elements.<br>• Flexibility in that educators can tailor the framework to the specific intervention design, factors, and context being studied.<br><br>**Strengths**<br>• Suited to guide rapid-cycle evaluation of the implementation of complex health care delivery interventions (multi-level contexts that can influence implementation.)<br>• Provides practical guide for systematically assessing potential barriers and facilitators in preparation for implementing an innovation or education program.

	Description	Strengths* and Limitations*
	engaging appropriate individuals in the implementation and use of the intervention, reflecting, and evaluating).	**Limitations**<br>• Many constructs may be overwhelming and challenging to select from.
**Developmental Evaluation (DE)** (31)(33)(32)	• Approach to evaluate the implementation of complex, emergent initiatives that are in the early stages of development.<br>• Embraces continuous reflection, learning, and adaptation.<br>• The process, based on reflective practices, is used mainly to respond to what initiatives are doing rather than attending to pre-determined outcomes.<br>• Examples of evaluation questions that can be addressed with DE include: 1) To what extent is or has the program helped to improve the care for people living with serious illnesses who experience challenges to accessing services? 2) To what extent has the program facilitated access of the service to patients or populations? 3) To what extent has the program connected patients to existing services? 4) To what extent has the program facilitated education and built capacity around palliative approaches to care for targeted patients or populations?	**Strengths**<br>• Particularly useful to evaluate community-directed interventions.<br>• Useful within changing and fluid contexts.<br>• Very adaptive (and embracing of) to nonlinear dynamics of real-world social interventions and complex interventions in complex contexts.<br>• Flexible and can be used for multiple purposes (e.g. development, evaluation, quality improvement)<br><br>**Limitations**<br>• Evaluation of impact is not explicit and unclear.<br>• Largely limited to programs in the relatively early stages of development.
**Active Implementation Framework**(34)	• Outlines five components for implementation and evaluation success integrated within four implementation stages. Within each of the four stages are a set of processes and activities.<br>• The five components for implementation and evaluation success include: (1) A Usable Intervention, (2) Implementation Stages, (3) Implementation Teams (e.g., implementation experts, intervention stakeholders, and intervention experts), (4) Implementation Drivers (e.g., competency drivers, organizational drivers, leadership drivers), and (5) Quality Improvement Cycles (e.g., Model For Improvement Plan Do Study Act (PDSA) cycles). Institute for Healthcare Improvement (IHI). Plan-Do-Study-Act (PDSA) Cycles. http://www.ihi.org/resources/Pages/Tools/PlanDoStudyActWorksheet.aspx<br>• The four implementation stages are: A) Exploration; B) Installation; C) Initial Implementation; and D) Full implementation.	**Strengths**<br>• Evidence-based synthesis of frameworks.<br>• Strong focus on program development, quality improvement, and understanding implementation to enhance success.<br>• Multipurpose<br><br>**Limitations**<br>• Impact analysis is not explicit.
**The RE-AIM (Reach, Effectiveness, Adoption, Implementation, and Maintenance) Framework**(38)(37)(39)	Specifically designed to evaluate the impact of community-based public health programs and interventions, and public services.<br>Consists of five evaluation dimensions:<br>• Reach (Represents the absolute number and proportion of individuals who are willing to participate in a given initiative and how representative participants are compared to the target population).<br>• Effectiveness (Describes the impact of an intervention or program on important outcomes, including potential positive and negative effects, quality of life, and	**Strengths**<br>• Well suited to assess community-based public health programs like Compassionate Community programs and public services, especially complex interventions and tasks as they often rely on multiple partners and input from different sources in often complex settings<br><br>**Limitations**

	Description	Strengths* and Limitations*
	economic outcomes)<br>• Adoption (absolute number and proportion of settings and intervention staff who are willing to initiate an intervention or program).<br>• Implementation (Refers to the degree to which the intervention or program staff deliver the initiative as intended as well as the related costs. Also refers to participants' use of the intervention's strategies or the program's services.<br>• Maintenance (Refers to the sustained delivery and effectiveness of the initiative)	• Impact analysis is not explicit.
**Evaluation of economic impact and return on investment (ROI)**		
**Health Technology Assessment (HTA)** (80)	• Used for systematic evaluations of properties, effects, and/or impacts of health technologies.<br>• Can also be used to evaluate health interventions; and social, economic, organizational, and ethical issues of a health intervention or health technology.<br>• Recognizes differences in the evaluation of costs and outcomes (e.g. perspectives of economic analysis such as society overall, a third-party payer, clinician, hospital, and/or patient)<br>• Calls for transparency of costing reporting; e.g. whether average costs (total or absolute costs) or marginal costs (outcome changes with changes in cost) are being used in the analysis.<br>• Requires that economic evaluation be integrated in overall evaluation of technology or intervention from outset.	**Strengths**<br>• a multidisciplinary process.<br>• Covers direct, intended consequences and indirect, unintended consequences.<br><br>**Limitations**<br>• Requires specialized expertise<br>• See "evaluation of impact" section above.
**Evaluations of processes, impact and/or return on investments**		
**Kirkpatrick's New World Model (NWKM)** (23)(43) **(includes drivers of change)**	• See "evaluation of impact" section above.	• See "evaluation of impact" section above.
**Logical Framework Approach (LFA)** (13)(82)	• Essentially an approach and tool for planning, managing and evaluating programs and projects. Provides a structure to help specify the components of a project, its activities, input and resources, anticipated challenges, influencing factors, indicators and for relating them to one another.<br>• Involves identifying strategic elements (inputs, outputs, outcomes, impact) and their causal relationships, indicators, and the assumptions or risks that may influence success and failure. It includes analyses of the need and stakeholders and determination of the programs overall and intermediate goals and activities and strategies to achieve these.<br>• These are displayed as a 4 X 4 matrix or more linear logical model graphic; referred to as the Logical Framework Matrix or Logframe. The first column captures the project's development pathway or intervention logic and usually includes, at the top, the overall goal and long-term goals, then the intermediate objectives,	**Strengths**<br>• Provides an overview of a project for purposes of planning, monitoring and evaluation.<br>• Guides project management, including implementation and monitoring<br>• Facilitates common understanding and better communication between stakeholder (decision makers, managers, workforce, partners, etc).<br>• Ensures that key questions are asked, and weaknesses are analyzed.<br>• Guides systematic and logical analysis of the inter-related key elements.<br>• Improves planning by highlighting linkages between

	Description	Strengths* and Limitations*
	followed by the outputs and then activities that are predicted to result in the objectives. Below or alongside are also listed the inputs, which include financial, human, and material needed for the program or project. The second and third columns summarize how the project's achievement will be monitored with the second column specifying the indicators (quantitative or qualitative measurements which provide a reliable way to assess changes connected to the intervention) and the third column listing the sources of data to provide evidence for the indicators. The final column lists the assumptions, which include the external factors or conditions outside of the project's control that are required to ensure success.<br>• Useful resource is The World Bank Logframe Handbook Guide: https://documents.worldbank.org/en/publication/documents-reports/documentdetail/783001468134383368/the-logframe-handbook-a-logical-framework-approach-to-project-cycle-management	project elements and external factors.<br><br>**Limitations**<br>• Tendency towards rigid application and risk of lack of flexibility to adjust to changing or new developments and opportunities.<br>• Can stifle innovation and adaptive management.<br>• Does not inform what and how to evaluate.<br>• LFA is only one of several tools to be used during project preparation, implementation and evaluation, and does not replace impact analysis (e.g. target-group analysis, cost benefit analysis)
**3-Wishes Project Evaluation Framework**(83)(61)	Not designed for evaluating education interventions, but well suited to do so.<br>Evaluates an intervention across four dimensions:<br>• Value (as experienced by friends, family, clinicians, managers, administrators),<br>• Transferability (related to signs of successful implementation beyond the original centre),<br>• Affordability (related to cost, per patient in this case); and<br>• Sustainability (related to project continuation beyond 1st year of evaluation).<br>Evidence for value, transferability and sustainability is largely through qualitative data collected through interviews and other artifacts (e.g. reports)	**Strengths**<br>• Ease of application<br>• Evidence gathered largely through interviews, focus groups and surveys (so practical)<br>• Aligns well with quality improvement philosophy and approach.<br><br>**Limitations**<br>• May not appeal to funders or decision-makers who place value on quantitative data and results.
**The Medical Research Council's Guidelines(22)**	Provides guidance on the development, evaluation and implementation of complex interventions to improve health. Although not specifically designed for education, it can be applied to education interventions.<br>Includes guidance on experimental and non-experimental methods for educators and researchers to select from.<br>It is divided into five main sections; each section provides key questions to use:<br>A) Developing an intervention;<br>B) Piloting and feasibility;<br>C) Evaluating the intervention;<br>D) Reporting; and<br>E) Implementation.<br>Provides frameworks to use experimental designs such as randomized trials, or alternatives if these are not feasible or appropriate. However emphasizes the need to evaluate other aspects of impact and of implementation.<br>Not framework or model as such, but set of guidelines.	**Strengths**<br>• Provides advice on how to select an experimental design to assess the impact of a complex health related intervention.<br>• Provides useful questions to inform development, evaluation and implementation.<br>• Recommends inclusion of an economic evaluation and inclusion of evaluation of implementation processes.<br><br>**Limitations**<br>• The guidelines or recommendations are not specific to education interventions.
**Realist Model**(30)(35)	Seeks to answer the questions:	**Strengths**

	Description	Strengths* and Limitations*
	• "What works for whom, in what circumstances and why?"' • "What kinds of educational interventions will tend to work, for what kinds of learners, in what kinds of contexts, to what degree, and what explains such patterns?" In seeking to answer these questions, it sets out to identify three fundamental components of an intervention; context, mechanism, and outcome (CMO). Aims to identify underlying causal mechanisms and how they work under varying conditions.	• Particularly suited to evaluating complex education interventions. • Flexible and stresses evaluation of interventions as they occur in the real world (not manipulated for experimental purposes). • Realist research uses mixed methods (qualitative and quantitative) approach to gather data and reach understanding; recognizes need to use different approaches for different questions, contexts and needs (urges judiciousness and flexibility) • Does not assume a simple cause-and-effect association <br>**Limitations** • Does not provide specific direction on domains or constructs to specifically evaluate
**Contribution Analysis (CA)** (24)(36)(81)	• Well suited to evaluate complex interventions • A theory-based approach to program evaluation that provides a systematic way to make credible causal claims when complex conditions exist. • Looks for full understanding of both how and why a program has been implemented. One searches for plausible descriptions of linkages between many program activities, their relationship to proximal and distal outcomes, and the underlying assumptions informing these connections. Understanding these helps to describe the "impact pathway", which helps to describe the theory of change. Requires multiple sources of evidence to describe the whole story over time, linkages, and impact • One approach to CA is by Mayne step model.(91) a. Step 1: Set out the cause-and-effect issue to be addressed. b. Step 2: Develops a postulated theory of change c. Step 3: Evidence is gathered for processes and outcomes (often using mixed methods approaches). d. Step 4: Assemble the contribution story e. Step 5: Educators and researchers seek out additional evidence (gathered through multiple methods; triangulation of findings enhances process). f. Step 6: The contribution story is revised and finalized.	**Strengths** • Particularly suited to evaluating complex education interventions. • Useful when an intervention is influenced by many external factors, has multifaceted objectives, and the impact unfolds over time. • Moves evaluators from attribution analyses (looking for direct causal relationships between the intervention and outcomes) to an approach that focusses on the question: "How much of a difference (or contribution) has the program made to the observed outcomes? <br>**Limitations** • Does not provide specific direction on domains or constructs to specifically evaluate
**Stake's Countenance Evaluation Model**(84)	The model has two major domains (or "countenances"); "Description" and "Judgment". "Description" relates to the characteristics of the education intervention, while "Judgement" relates to making a ruling as to whether the program attained standards or not. The Model then describes three phases in each of the two domains, namely the "antecedent" phase (the conditions and circumstances existing prior to the education	**Strengths** • Highlights the need to evaluate and understand connections (convergence or divergence) between what was intended and what actually materialized. • The matrix helps identify points at which instruments or other methods (such as interviews) should be used to

	Description	Strengths* and Limitations*
	intervention), the "transaction" phase (successive engagements of encounters during the actual instruction), and the "outcome" phase (the effects of the instructional experience).<br>The domains, phases, and streams collectively give rise to a matrix with 12 cells, 6 for the "Description" domain and 6 for the "Judgement" domain.	collect data.<br><br>**Limitations**<br>• Complicated to navigate and execute.<br>• Impact analysis is not explicitly highlighted.
**LENSES, PARADIGMS AND OTHER APPROACHES**		
**Quality Improvement (QI) and the Triple and Quadruple Aims**(46)(48)(47)(92)	The overarching goals of undertaking continuous QI are expressed in the Triple and Quadruple Aims.<br>**Triple Aim:** (47)<br>• Improve the patient experience,<br>• Achieve better health outcomes (including quality of care and patient safety),<br>• Improve efficiencies (and lowering health care costs).<br>**Quadruple Aim and Quintuple**(46)(48)<br>• As for Triple Aim, PLUS improve the care provider experience, and these four goals are collectively referred to as the Quadruple Aim.<br>**Institute of Medicine's six dimensions of quality**(49)<br>• Timeliness, efficiency, equitability, safety, effectiveness and patient-centredness.<br>The aims and dimensions provide constructs with which to approach the impact of education interventions on patients, care, the health care system and healthcare providers.	
**The Pragmatism Paradigm**(50)(85)	Pragmatism does not commit itself to a specific epistemology or reality. Rather, methods are selected on their relevance to the issue or program being evaluated, or aspect of a program being evaluated.	
**Social Network Analysis (SNA)** (53)(51)(52)	Social networks are important at individual and organization levels, particularly in terms of spreading knowledge, expertise, experiences, and innovations. Different factors and dynamics determine the structure and strength of the network(s). These connections, or lack thereof, can be studied and can provide insights into the spread and scale-up of an intervention or innovation. Analysis can be carried out via phenomenological, ethnographic, or diagramming techniques.	
**Theories of Change**	Some experts advocate for the importance of identifying theories that underpin an education program as these inform the design of the interventions, their implementation and spread, and ultimately their evaluation, especially if using a Realist model or approach.(35)<br>Examples of these theories include:<br>• Design:<br>○ Social constructivism, Cognitive flexibility theory and Collaborative Learning(93)(94)<br>• Change and Dissemination:<br>○ Roger's Diffusion of Innovation theory). (95)(96)<br>○ Theory of Planned Behaviour (TPB). (86)(87)(88)<br>○ Michie et al's Behaviour Change Wheel(89)<br>○ The COM-B-System(90)	
**Participatory Action Research (PAR) and Community-Based Participatory Research (CBPR).** (56)(57)	While not an evaluation model or framework per se, participatory action research and CBPR provide approaches that can be useful in some evaluation needs, goals and contexts. PAR is an approach to research that emphasizes participation and action. It seeks to understand the world by trying to change it, collaboratively, and following reflection. PAR emphasizes collective inquiry and experimentation grounded in experience and social history. PAR practitioners make a concerted effort to integrate three basic aspects of their work: participation, action, and research.	

	Description	Strengths* and Limitations*
(54)(55)(56)(40)		
